# Detection and characterisation of canine astrovirus, canine parvovirus and canine papillomavirus in puppies using next generation sequencing

**DOI:** 10.1038/s41598-019-41045-z

**Published:** 2019-03-14

**Authors:** Tarka Raj Bhatta, Anthony Chamings, Jessy Vibin, Soren Alexandersen

**Affiliations:** 1 Geelong Centre for Emerging Infectious Diseases, Geelong, VIC 3220 Australia; 2 Deakin University, School of Medicine, Geelong, VIC 3220 Australia; 3 Barwon Health, University Hospital Geelong, Geelong, VIC 3220 Australia

## Abstract

Gastroenteritis in young animals is a clinical presentation with many infectious and non- infectious aetiologies. We used next generation sequencing (NGS) to investigate the possible infectious causes of gastroenteritis in puppies from a dog kennel in Victoria, Australia. The near complete genome of a canine astrovirus was obtained from pooled faecal samples, and was found to be 94.7% identical with a canine astrovirus detected in the United Kingdom in 2012. The phylogenetic analysis of the capsid gene found similarities to those of canine astroviruses identified in Italy in 2005 and in UK and Hungary in 2012, but distant from that of a canine astrovirus previously identified in Australia in 2012. Thus, different serotypes of canine astrovirus are likely circulating in Australia. The close relationship to European astroviruses also suggested that there had been recent movements of ancestor canine astroviruses between Australia and Europe. NGS also detected other infections in the puppies including several canine papillomaviruses and a canine parvovirus (vaccine strain) as well as a very low level of campylobacter. Canine astrovirus was the probable cause of diarrhoea in these puppies, with the possible involvement of campylobacter bacteria. NGS was effective as a non-targeted method to determine the likely infectious cause of gastroenteritis.

## Introduction

Canine infectious gastroenteritis is one of the common reasons for presentation to a veterinary clinic and hospitalization. It can be challenging for the veterinarian to determine the actual causative agent responsible for diarrhoea due to the diverse array of pathogenic agents which could be responsible including viruses, bacteria, or protozoans^1,2^. Viruses are a common aetiology, with one or more detected in 40–60% of cases of gastroenteritis in dogs in several studies^2–4^.

Detection of gastroenteritis causing viruses like canine astrovirus, canine parvovirus, canine coronavirus, canine rotavirus and canine distemper can be performed using targeted rapid antigen detecting enzyme immunoassays or Polymerase Chain Reaction (PCR) assays^5–10^. But targeted detection techniques may not be ideal if novel strains or viruses are involved^11^ or if multiple viruses are present^12^.

Because of the advancement in the Next Generation Sequencing technology (NGS), it has become easier to detect novel and multiple pathogens in a single test. However, its application to the diagnosis of the causes of gastroenteritis in dogs has been limited to date^13,14^. However, when it has been used, it has detected a diverse range of viruses in dogs with diarrhoea including canine sapovirus, canine kobuvirus, canine parvovirus, canine astrovirus, canine rotavirus and canine coronavirus in dogs with acute diarrhoea^13,14^.

The study described here focused on samples from a single, relatively large dog kennel with Labrador retrievers located in Victoria, Australia. The kennel has around 20 dogs including breeding females and males, a variable number of young dogs, older retired dogs as well as visiting dogs. This kennel consequently represent a very active environment with dogs coming and going, potentially introducing new pathogens or allowing amplification of pathogens already present. The dogs at the kennel are very well taken care of and receive routine vaccinations, including first vaccination of puppies at 6 weeks of age, and other veterinary treatments provided by a local veterinary hospital. In September 2017 the kennel had 3 litters (around 30 puppies) born within the same week, and while the puppies initially did well, the puppies in one of the litters, and born by a female dog having been stationed away from the kennel at a private home until only one week before giving birth, showed relatively severe signs of gastrointestinal disease when approximately 5 weeks of age. Several of the puppies were examined at the local veterinary hospital and tentatively diagnosed with, and treated for, campylobacter infection. The puppies recovered well and at the age of approximately 7 weeks when they were in good health again, we took environmental samples as detailed below for analysis by next generation sequencing. The aim was to determine if next generation sequencing could be used to identify the possible cause(s) of the gastroenteritis.

## Results

Based on the initial BLASTN and BLASTX analysis of the next generation sequencing (NGS) reads, 3 viruses of interest were identified and became the focus for further analysis: canine astrovirus and canine parvovirus from the pooled faecal sample and canine papillomavirus from the pooled and individual saliva samples. A few reads of canine astrovirus were also obtained from each of the individual saliva samples. However, significantly more reads were obtained from the pooled faecal sample and the near complete genome was able to be constructed from this sample alone.

### Molecular and phylogenetic characterization of canine astrovirus

The near full-length sequence of a canine astrovirus was obtained from the pooled canine faecal sample DF-1-2-AUS-2017. The obtained sequence DF-BC15-CAV-AUS-2017 was 6612 nucleotides long and was derived from reads mapped to the canine astrovirus reference sequence KP404149-CAV-Gillingham-UK-2012^15^ with a coverage depth ranging from 22 to 6375 and a mapping quality threshold of 32 and above. The assembled genome included 39 nucleotides of the 5′ untranslated region (UTR), missing only 6 nucleotides from the very 5′ end, a 2670 nucleotides long open reading frame 1a (ORF1a), a 1530 nucleotides long ORF1b, a 2505 nucleotides long ORF2, an 82 nucleotides long 3′UTR and a 17 nucleotides long polyA-tail.

**Figure 1:**
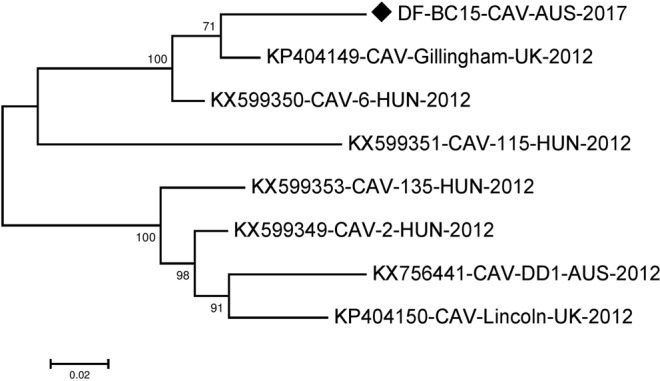
Phylogenetic analysis of the canine astrovirus near-complete genome. The nucleotide sequences were aligned and analysed using the maximum likelihood method in MEGA 7.0^43^ using the General Time Reversible (GTR + G + I)^50^ model with a bootstrapping of 1000 replicates. The analysis involved 8 sequences of the canine astrovirus genome including canine astrovirus sequence DF-BC15-CAV-AUS-2017 from the dog samples. The numbers at nodes represent bootstrap values. Branch lengths are scaled according to the numbers of nucleotide substitutions per site.

**Figure 2:**
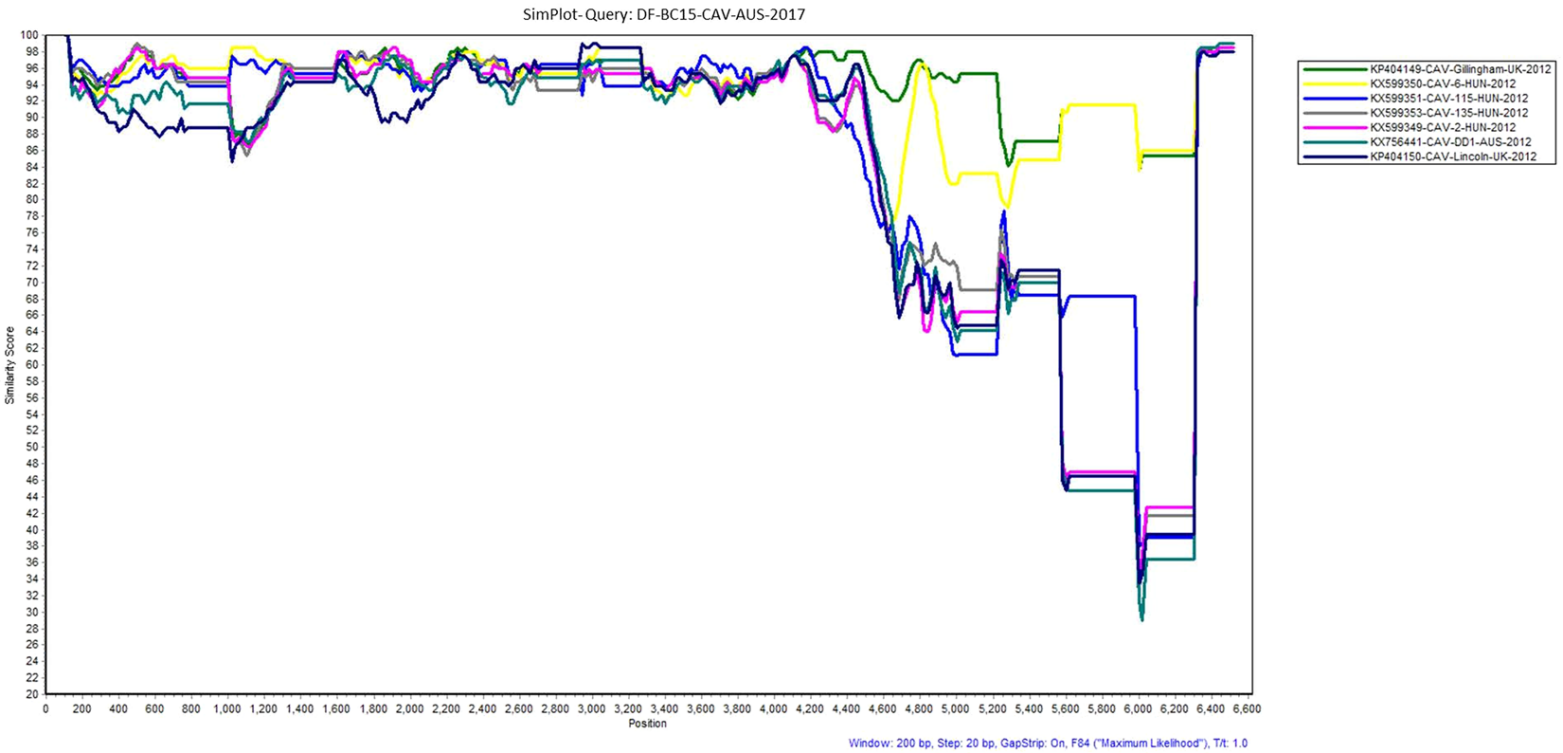
Similarity plot generated in SimPlot using the canine astrovirus sequence (DF-BC15- CAV-AUS-2017) from the dog samples as the query sequence against seven other reference sequences from NCBI Genbank. Divergence between DF-BC15-CAV-AUS-2017 and each reference sequence over the 6612 nucleotides genome using a 200 nucleotides sliding window at 20 nucleotides intervals and the F84 distance^46^ model with the maximum likelihood method. Percentage identities at each analysis point were plotted on a line chart. For similarity plot analysis, the *y*-axis shows the percentage similarity between the parental sequences and the query sequence. Different colors represent a different reference sequence.

For phylogenetic analysis of our near full-length canine astrovirus sequence, we initially selected the seven most closely related near full-length canine astrovirus sequences from dogs and one mamastrovirus sequence from a fox available in the NCBI Genbank summarized in Table S1. KY765684-CTAV-BRA-2015^16^ from a fox did not fall in any cluster with the astroviruses from domestic dogs and was therefore not included in the phylogenetic analyses shown. The generated maximum likelihood (ML) phylogenetic tree (Fig. 1) indicated that our sequence DF-BC15-CAV-AUS-2017 was more related to KP404149-CAV-Gillingham-UK-2012 and KX599350-CAV-6-HUN-2012^15,17^ as compared to those in the cluster with KP404150-CAV-Lincoln-UK-2012^15^, KX756441-CAV-DD1-AUS- 2012^14^, KX599353-CAV135-HUN-2012^17^ and KX599349-CAV-2-HUN-2012^17^. KX599351-CAV-115-HUN-2012^17^ appeared to fall somewhat in between these two clusters (Fig. 1). The SimPlot analysis (Fig. 2) did largely agreed with the clustering of sequences mentioned above. The majority of differences between sequences were located in the ORF2 (capsid) region downstream of nucleotide 4009 and may have involved recombination or possibly evolution/selection of different capsid genotypes/serotypes.

### Analysis of the non-structural protein open reading frame (ORF1a) and the RNA dependent RNA polymerase (RdRp) open reading frame (ORF1b)

**Figure 3:**
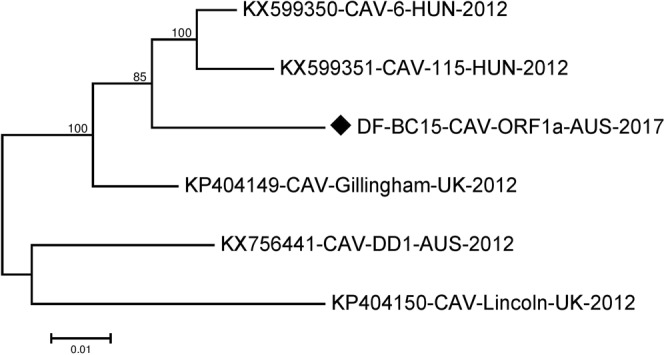
Phylogenetic analysis of the nucleotide sequence of the canine astrovirus ORF1a region. The nucleotide sequence identity of DF-BC15-CAV-ORF1a-AUS-2017 was analysed using the maximum likelihood method in MEGA 7.0^43^ using the General Time Reversible (GTR + G)^50^ model and with a bootstrapping of 1000 replicates. The analysis involved 6 sequences of canine astrovirus ORF1a region. The numbers at each node represent the bootstrap values. Branch lengths are scaled according to the numbers of nucleotide substitutions per site.

**Figure 4:**
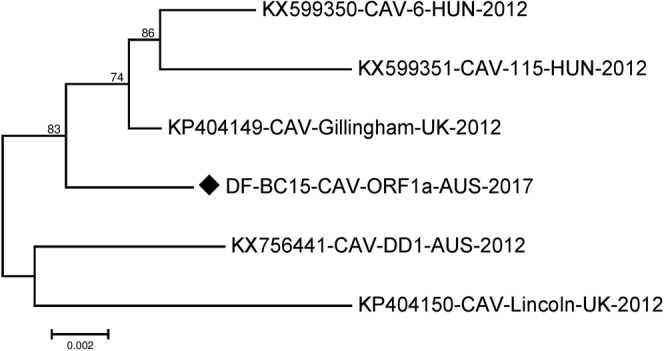
Phylogenetic analysis of the amino acid sequence of canine astrovirus ORF1a region. The amino acid sequence of DF-BC15-CAV-ORF1a-AUS-2017 was analysed using the maximum likelihood method in MEGA 7.0^43^ using the Jones-Taylor-Thornton (JTT + G)^51^ model and with a bootstrapping of 500 replicates. The analysis involved 6 sequences of the canine astrovirus ORF1a region. The numbers at nodes represent bootstrap values. Branch lengths are scaled according to the numbers of amino acid substitutions per site.

The ORF1a sequence was 2670 nucleotides long and encodes for an 889 amino acid polyprotein. For the phylogenetic analysis of the ORF1a we could unfortunately not include all sequences mentioned in Table S1 because some of the HUN-2012 sequences had what appear to be insertions/deletions, or possibly sequencing artefacts that would make phylogenetic inference unreliable. Consequently, for the ORF1a we only compared our sequence DF-BC15-CAV-ORF1a-AUS-2017 to five other related canine astrovirus sequences. This analysis suggested that in this part of the genome our sequence clustered with KX599350-CAV-6-HUN-2012^17^, KX599351-CAV-115-HUN-2012^17^ and KP404149- CAV-Gillingham-UK-2012. KX756441-CAV-DD1-AUS-2012 and KP404150-CAV-Lincoln-UK-2012 were more distantly related (Figs 3 and 4 and Table S2).

**Figure 5:**
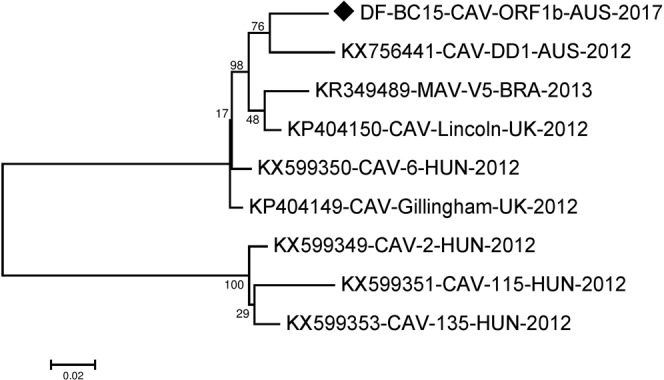
Phylogenetic analysis of the nucleotide sequence of canine astrovirus ORF1b region. The nucleotide sequence of DF-BC15-CAV-ORF1b-AUS-2017 was analysed using the maximum likelihood method in MEGA 7.0^43^ using the Tamura-Nei model (T93 + G)^52^ model and with a bootstrapping of 1000 replicates. The analysis involved 9 sequences of the canine astrovirus ORF1b region. The numbers at nodes represent bootstrap values. Branch lengths are scaled according to the numbers of nucleotide substitutions per site.

**Figure 6:**
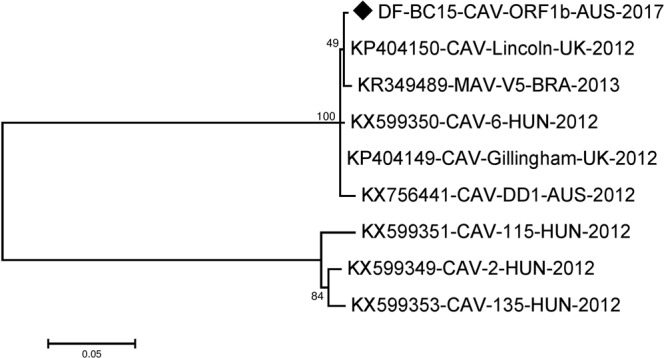
Phylogenetic analysis of the amino acid sequence of canine astrovirus ORF1b region. The amino acid sequence of DF-BC15-CAV-ORF1b-AUS-2017 was analysed using the maximum likelihood method in MEGA 7.0^43^ using the Jones-Taylor-Thornton (JTT + G)^51^ model and with a bootstrapping of 500 replicates. The analysis involved 9 sequences of the canine astrovirus ORF1b region. The numbers at nodes represent bootstrap values. Branch lengths are scaled according to the numbers of amino acid substitutions per site.

The ORF1b sequence was 1536 nucleotides long and encodes a 511 amino acids long RNA dependent RNA polymerase. The region was well conserved amongst the canine astroviruses when compared to other regions as evident by the SimPlot analysis (Fig. 2). For further analysis we included an additional canine astrovirus sequence from Brazil in year 2013 (KR349489-MAV-V5-BRA-2013)^18^ together with the other reference sequences, except KY765684-CTAV-BRA-2015^16^ (Table S1). The phylogenetic analysis indicated that the sequences included here fell into 2 clusters with KP404150-CAV-Lincoln-UK-2012, KR349489-MAV-V5-BRA-2013, KX599350-CAV-6-HUN-2012, KP404149-CAV-Gillingham-UK-2012, KX756441-CAV-DD1-AUS-2012 and our DF-BC15-CAV-ORF1b-AUS-2017 in one cluster while KX599349-CAV-2-HUN-2012, KX599351-CAV-115-HUN-2012 and KX599353-CAV-135-HUN-2012 fell in a different cluster based on phylogenetic trees of both nucleotide and amino acid sequences (Figs 5 and 6).

### Analysis of the capsid protein open reading frame (ORF2)

**Figure 7:**
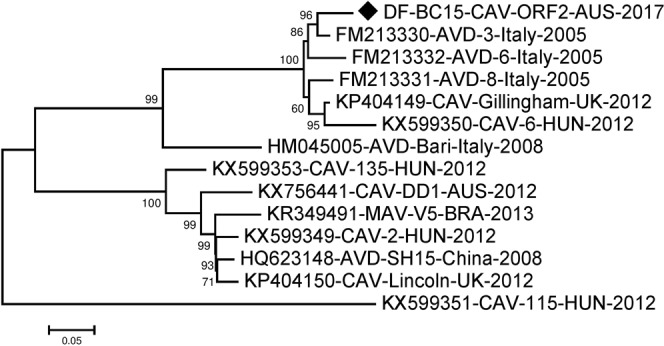
Phylogenetic analysis of nucleotide sequence of canine astrovirus ORF2 region. The nucleotide sequence of DF-BC15-CAV-ORF2-AUS-2017 was analysed using the maximum likelihood method in MEGA 7.0^43^ using the General Time Reversible (GTR + G + I)^50^ model and with a bootstrapping of 1000 replicates. The analysis involved 14 sequences of the canine astrovirus ORF2 region. The numbers at nodes represent bootstrap values. Branch lengths are scaled according to the numbers of nucleotide substitutions per site.

**Figure 8:**
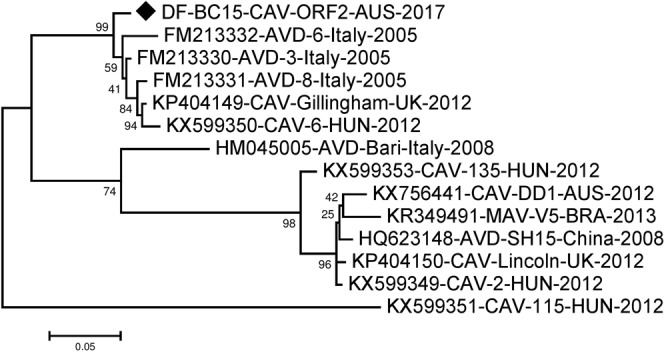
Phylogenetic analysis of amino acid sequence of canine astrovirus ORF2 region. The amino acid sequence identity of DF-BC15-CAV-ORF2-AUS-2017 was summarized using the maximum likelihood method in MEGA 7.0^43^ using the Jones-Taylor-Thornton (JTT + G)^51^ model and with a bootstrapping of 500 replicates. The analysis involved 14 sequences of the canine astrovirus ORF2 region. The numbers at nodes represent bootstrap values. Branch lengths are scaled according to the numbers of amino acid substitutions per site.

The ORF2 sequence was 2505 nucleotides long and encodes for an 834 amino acids long capsid polyprotein. For analysis of ORF2 we included additional sequences as more were available in the NCBI Genbank. These included three sequences from Italy in 2005 and one from 2008, and another sequence from China in 2008. Alignment and phylogenetic analysis using MEGA showed a nucleotide identity between 76.65–95.29% and amino acid identity between 78.61%-97.36%. Our sequence DF-BC15-CAV-ORF2-AUS-2017 was found to be closest to FM213330-AVD-3-Italy-2005^6^ having 118 nucleotides differences (95.29% identity) and 22 amino acids differences (97.36% identity). Four other sequences that were in the same cluster (Figs 7 and 8). Most other sequences fell into another distinct cluster with KP404150-CAV-Lincoln-UK-2012, while HM045005-AVD-Bari-Italy-2008^5^ fell in between the two clusters and KX599351-CAV-115-HUN-2012 appeared to be somewhat different from all other sequences (Figs 7 and 8).

**Figure 9:**
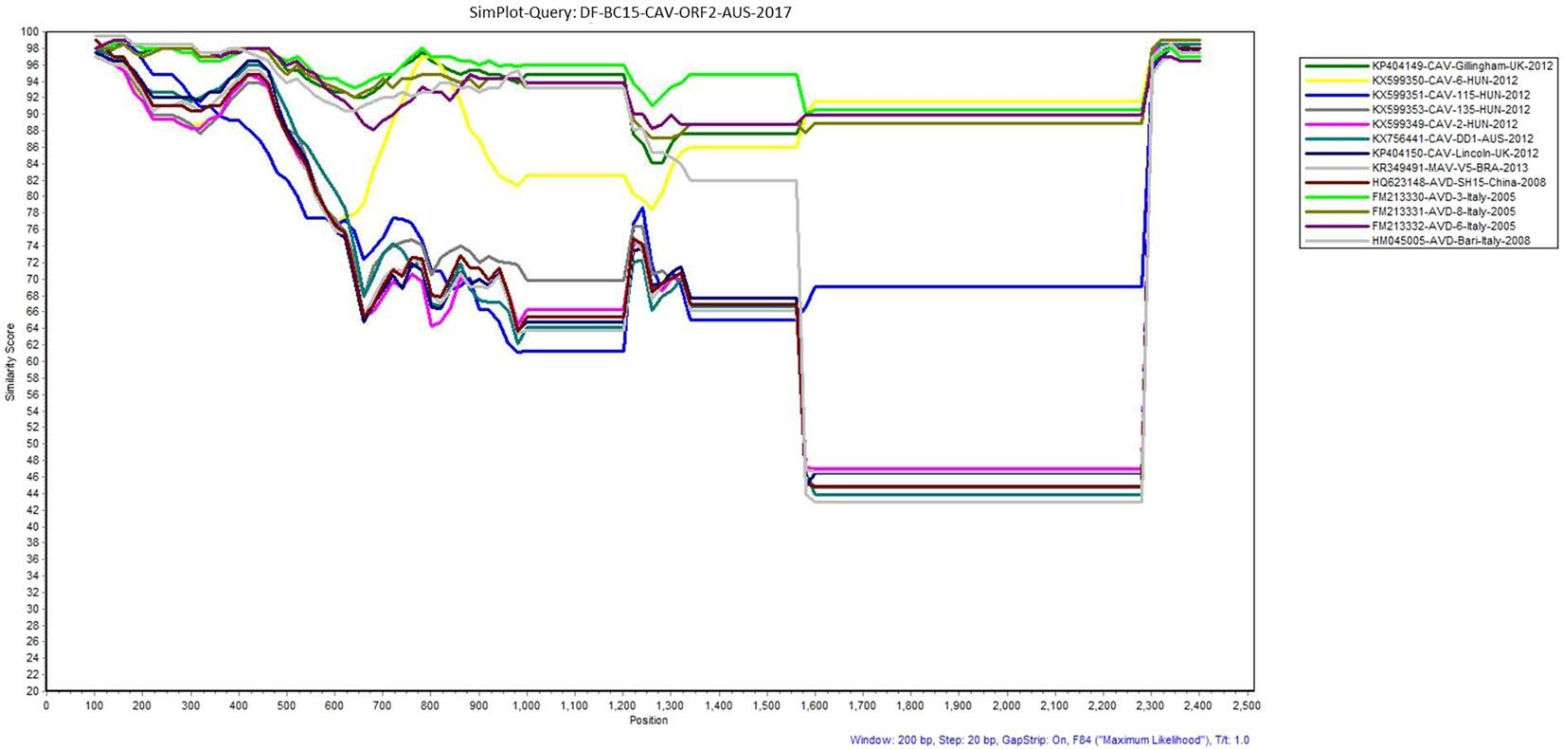
Similarity plot generated in SimPlot using the DF-BC15-CAV-ORF2-AUS-2017 sequence as the query sequence against thirteen other reference sequences. Divergence between DF-BC15-CAV-ORF2-AUS-2017 and the reference virus sequences over the 2505 nucleotides of ORF2 using a 200 nucleotides sliding window at 20 nucleotides intervals and the F84 distance^46^ model with the maximum likelihood method. Percentage identities at each analysis point were plotted on a line chart. For similarity plot analysis, the y-axis shows the percentage similarity between the reference sequences and the query sequence. Different colours are indexed for different reference sequences.

Simplot analysis of DF-BC15-CAV-ORF2-AUS-2017 against the other canine astrovirus sequences showed the central region of the capsid protein coding region to be most different (Fig. 9), which is consistent with findings of other investigators^7^. The ORF2 sequences included in the phylogenetic analysis fell into two major clusters which could represent possible serotypes. If so, DF-BC15-CAV-AUS-2017 may be more antigenically related to canine astroviruses from Europe than the only other characterized Australian canine astrovirus^3^ (Figs 7–9).

### Detection of canine astrovirus by PCR in samples from individual dogs

Astrovirus nucleic acid was detected in both of the two faecal samples collected from puppies in November 2017 using the ORF1b PCR. Sanger sequencing of the PCR products found that the sequence of the amplicons was 100% identical to the sequence obtained by NGS in the targeted region. In contrast, all seven faecal samples collected in January 2018 were all negative for astrovirus by PCR.

### Detection and molecular characterization of canine parvovirus

We were able to generate 7 relatively short consensus sequences from the NGS reads from the dog faecal sample DF-1-2-AUS-2017 when mapped to the reference canine parvovirus genome M38245-CPV-b-USA-1990^19^. The number of reads were much fewer than for the astrovirus described above, but consensus sequences including 324 nucleotides (coverage 7–141) of the 3′-NCR-NS1, 453 nucleotides (coverage 7–235) and 319 nucleotides (coverage 18–174) of NS1, 458 nucleotides (coverage 31–735) of VP1, 565 nucleotides (coverage 18–229) and 621 nucleotides (coverage 18–403) of VP2 and a 140 nucleotides (coverage 18–51) of the 3ˈ-non-coding region were assembled from the mapped reads [see Table S3 for details].

To look into whether the canine parvovirus sequences found in the dog sample may have been derived from vaccination (the puppies were vaccinated just one week before sampling), further alignments were made with additional sequences, including vaccine virus sequences, collected from NCBI Genbank or from published literature^20^. This alignment showed that our sequences matched 621/621 (100%) nucleotides with the Protech-CPV-2-C3-Vac-AUS-2016^20^ in the VP2 region between nucleotides 3593–4213, but only 615/621 (98.71%) nucleotides with the Canigen-CPV-2-C3-Vac-AUS-2016^20^ indicating that our sequence might have come from the Protech-CPV-2-C3-Vac-AUS-2016 vaccine. Comparing the 3ˈ-non-coding sequence from our sequence DF-BC16-CPV-AUS-2017 to the published Protech-CPV-2-C3-Vac-AUS-2016 vaccine sequence, a single mismatch was noted at position 4844.

### Detection and molecular characterization of canine papillomavirus

Eight relatively short consensus sequences were able to be generated when reads from the pooled saliva sample were mapped with a high mapping quality of 90 against canine papillomavirus 17 (Genbank accession KT272399)^21^, although the number of mapped reads was low (coverage 2–12). The sequences assembled included regions of E1, E2, L2 and L1. Two non-coding regions were also partially sequenced i.e. the non-coding sequence between the E2 and L2 region (291 nucleotides) and that between L2 and L1 (16 nucleotides). These high quality sequences were further used for doing Clustal W alignment. Our sequences DS- BC18-CPapV-AUS-2017 showed the highest identity ranging from 99.5% to 100% with the reference sequence KT272399-CPapV-17-NZ-2014^21^ [See details in Table S4]. The sequence KT272399-CPapV-17-NZ-2014 was isolated from an oral squamous cell carcinoma from a dog (Labrador retriever) in New Zealand in 2014 and was designated canine papilloma virus 17 (CPapV)17^21^.

To see if the individual dog saliva samples also contained detectable canine papillomavirus, we processed the saliva samples individually in a second NGS run at a mapping quality of 80 and with a coverage of 2–18. One sample had canine papillomavirus 8 only, while another sample had the canine papillomavirus 17 seen in the pooled sample and other reads mapped to canine papillomavirus 4. The other 2 saliva samples did not have detectable papillomavirus virus in them. Three short consensus sequences were generated for canine papillomavirus 8 having a total length of 903 nucleotides from the individual sample DS-4-AUS-2017 after mapping against HQ262536-CPapV-8-Swiss-2010^22^. Regions E1 (224 nucleotides), E2 (395 nucleotides) and L1 (284 nucleotides) were obtained. Our sequences were found to be 100% similar with the reference sequence HQ262536-CPapV-8-Swiss-2010. Similarly, 4 consensus sequences were generated for canine papillomavirus 4 with a total length of 683 nucleotides in total (E7 140 nucleotides, E1 167 nucleotides and E2 114 and L2 262 nucleotides). EF584537-CPapV-4-Swiss-2007^23^ was found to be most similar genome compared to our sequences with 99.6–100% identity. The detail about sequence comparison can be found in Table S5.

## Discussion

NGS was an effective technique to detect and characterize possible causes of gastroenteritis or other infectious diseases as here presented for a litter of puppies. The puppies were sick with diarrhoea/intestinal problems and *Campylobacter* was tentatively diagnosed at the local veterinary hospital. Samples were obtained 10–14 days after resolution of clinical signs, and a reasonably high level of canine astrovirus reads was detected. This may have been the cause of the clinical disease, however a few reads matching bacterial 16S ribosomal RNA belonging to the genus *Campylobacter*, including the species *Campylobacter canadensis* and *Campylobacter upsaliensis*, were also identified among the NGS reads (data not shown). Both of these bacteria are known to cause enteric campylobacteriosis in companion animals and possibly in people^24–26^. Therefore, the severe clinical disease seen in this case may have been as a result of co-infection with astrovirus and *Campylobacter*, although it is also possible that the astrovirus infection only occurred after an initial *Campylobacter* infection. The fact that astrovirus was only detected by PCR shortly after clinical disease was observed, and not two and half months later, indicates that this virus was not continually circulating in dogs on the property, and that its presence was at least temporally associated with the clinical disease.

The presence of astrovirus sequences also in a puppy without diarrhoea suggests that astrovirus infection alone may not have been the sole reason for the gastroenteritis or that a difference in immune status may play a role. There are several reports in the literature where astrovirus infection has been associated with gastrointestinal disease in dogs in Australia^14^, UK^15^, China^7^, Korea^27^, Brazil^28^, Italy^5^ and Japan^29^, and some reports where it was detected in the absence of clinical gastroenteritis^17,30^. Similarly, in humans, astrovirus is one of the major cause of gastroenteritis in children^31^, yet asymptomatic infections have also been observed^32,33^. In this study, we speculate that the astrovirus positive puppies may have differed in their neutralizing antibody levels to astrovirus obtained from their mothers, or in the possible co-infections such as *Campylobacter spp*. present with the astrovirus, resulting in the difference in their disease status. The low level of parvovirus identified in the puppies appeared to be residual vaccine virus, and was therefore unlikely to be involved in the clinical disease. Ideally, sampling during the episode of gastroenteritis would have allowed better determination of the infections present at the time of the clinical signs, however the value of NGS to detect co-infections is clearly demonstrated here.

Phylogenetic analysis of our near full-genome sequence of canine astrovirus (DF-BC15- CAV-AUS-2017) with other reference sequences showed that it was most closely related to KP404149-CAV-Gillingham-UK-2012, identified in the United Kingdom five years earlier. Analysis of the ORF2 region, which is the most variable part of the genome, showed that the closest related sequence was FM213330-AVD-3-Italy-2005, identified in Italy about 12 years earlier. Australia and the United Kingdom/Europe are geographically remote, however dogs do move between these countries, and dog semen is imported to and from Australia for breeding programs^34,35^. It is therefore possible that an ancestor canine astrovirus was transported to/from Australia through one of these routes. Astrovirus is not currently screened for routinely as part of export/import quarantine requirements and therefore any movement of the virus between countries would have gone undetected at the time.

The only Australian canine astrovirus sequence available in Genbank at the time of writing was KX756441-CAV-DD1-AUS-2012 and this clustered into a different group to DF-BC15- CAV-AUS-2017 when the capsid (ORF2) gene sequences were compared. This suggested that these viruses possibly belonged to different serotypes. These viruses also fell into different phylogenetic clusters when other sections of the genome were compared. These viruses were identified from dogs in the same geographical region, and this suggests that there is likely a significant diversity of canine astroviruses circulating in Australia. However it would be necessary to culture and isolate this virus *in vitro* to characterize its serotype, but the adaptation of some astroviruses to grow in cell culture can be challenging^36,37^ and hence it was not done as part of this study.

We also detected a low level of canine parvovirus in the samples from the puppies, and sequence analysis suggested that was a CPV2 vaccine virus given to the puppies one week before sampling.

In the pooled saliva samples, sequences mapping closely to canine papillomavirus 17 were observed. The canine papillomavirus 17 sequence found in the puppies’ saliva samples were very closely related to KT272399-CPapV-17-NZ-2014, a virus identified in New Zealand in 2014^21^. Older puppies from the kennel in our study had a history of benign cutaneous warts which subsequently resolved. Interestingly, there was also a history of dogs from New Zealand being used in the breeding program at this kennel, which may explain the presence of a highly similar papillomavirus to that seen in New Zealand. There was also evidence that canine papillomavirus types 4 and 8 were also present in some of these puppies.

This study has shown that NGS can be useful in veterinary diagnostics in both detecting co- infections, and in providing potential useful epidemiological information as to the possible origin of those infections. However, the main current limitation is the time taken to perform the data analysis from a single sample. As computational power increases, it is likely that these results could be made available in a clinically relevant time frame and therefore become part of routine laboratory testing.

## Materials and Methods

### Samples

Samples for our study were collected with the consent of the owner in early November 2017, and consisted of faecal samples collected from the ground and saliva samples collected from empty food bowls. Faecal samples were collected immediately after being deposited by individual puppies, while saliva samples were taken by swabbing food bowls immediately after a litter of puppies had emptied and licked the bowls while deposited saliva was fresh. The studies described here were performed in accordance with all relevant guidelines and regulations and as only environmental samples were included, do not require animal ethics approval. Swabs were placed in Universal Transport Medium (UTM) and stored on ice overnight. The following morning, swabs were aliquoted and frozen at minus 80 °C until analysis. The samples analysed in the present study included faecal samples from 2 puppies (1 from the group of puppies with prior gastrointestinal disease and 1 from a group of puppies from the litters without signs of disease), and saliva samples from the same two groups of puppies with and without prior disease, respectively. To reduce initial NGS testing costs, the 2 faecal samples were combined and the 4 food bowl swabs (saliva samples) combined, so that the study included a single pooled faecal sample DF-1-2-AUS-2017 and a single pooled saliva sample DS-3-4-5-6-AUS-2017. The four saliva samples were also later processed separately for testing with NGS to better investigate the viruses present in the individual dogs. In mid-January 2018, two and half months after the initial sampling of the puppies, the kennel was revisited and individual faecal samples were collected from 7 dogs aged between 3 days and 2 years old to determine if a canine astrovirus, present at the initial sampling, was still circulating there.

### Virus enrichment, Nucleic acid extraction and cDNA synthesis

For NGS, the samples were processed, and virus particles enriched using a protocol optimised in our laboratory described previously^38^. Briefly, the samples were homogenised at 25 Hz for 2 min followed by centrifugation at 17000 g for 3 min and filtered using a 0.8 um Polyethersulfone (PES) Spin-column filter. The sample was then divided into two, one being ultracentrifuged at 178,000 g for 1 h and the other not ultracentrifuged. Both were then treated with benzonase and micrococcal nuclease for 2 h to enrich for nucleic acids protected in virus particles^38^. Nucleic acids (both DNA and RNA) were extracted using QIAamp Viral RNA Mini Kit (Qiagen). An initial RNA denaturation step of 95 °C for 3 min followed by snap-cooling in an ethanol-ice bath (minus 20 °C) was carried out before cDNA synthesis and DNA amplification using the SeqPlex RNA Amplification Kit (Sigma) as per the protocol described^38^. For PCR, cDNA synthesis of nucleic acids extracted from the individual faecal samples were conducted by using SuperScript™ VILO™ Master Mix (Invitrogen) at 25 °C for 10 min, 42 °C for 60 min, 85 °C for 5 min and hold at 4 °C in Applied Biosystems ProFlex PCR system (Thermofisher Scientific).

### Next Generation Sequencing (NGS)

Library preparation was carried out using the Ion Plus Fragment Library Kit (Thermo Fisher Scientific), Ion Xpress Barcode Adapters 1–96 Kit (Thermo Fisher Scientific), Agencourt AMPure XP kit (Beckman Coulter) and Ion Library TaqMan™ Quantitation Kit (Thermo Fisher Scientific) as per the manufacturer’s protocols. Pooled barcoded libraries were then loaded onto Ion 540 Chips using the Ion Chef Instrument. The loaded 540 chips were run on the Ion Torrent S5XL System (Thermo Fisher Scientific) as per the manufacturer’s protocols. Sequencing was performed at the Geelong Centre for Emerging Infectious Diseases (GCEID), Geelong, Victoria, Australia. The dog samples generated approximately 9.6 million reads for DF-1-2-AUS-2017 and 8.7 million reads for DS-3-4-5-6- AUS-2017, while the individual 4 dog saliva samples each generated 3.4 to 4.2 million reads.

### Detection of virus in the samples

The sequence data generated for each sample were analysed further for the detection of viruses using BLASTN^39,40^ query with an e-value cut-off score of 1 × 10^−10^ and BLASTX^39,40^ query with an e-value cut-off of 1 × 10^−10^ against a local database created from available virus sequences from the NCBI GenBank genetic sequence database (downloaded May 2018) as described previously^38^. Based on the number of high quality reads (initially Q > 20) matched to the reference viruses, and coverage of the reads across the reference genomes, we focused our analysis on 3 viruses of interest present in the samples: canine astrovirus, parvovirus and papillomavirus. The TMAP plugin^41^ from the Ion Torrent suite was used for mapping the NGS reads using selected reference genomes of these viruses from NCBI Genbank [see Table S6 for details] which were identified as most similar to the viruses in the dog samples. Near full or partial consensus sequences were generated from the mapped sequences using Integrative Genomics Viewer software (IGV) (Broad Institute, MA, USA)^42^ with a mapping quality score of 32 or higher and a coverage depth of at least 2 with further details noted in Results.

### Phylogenetic analysis

The virus consensus sequences were compared with the online NCBI Genbank using BLASTN and the closest related sequences selected for further analyses using MEGA 7 software^43^. For the canine parvovirus 2 (CPV2) comparisons, we also added the available sequences of the CPV2 Protech C3 Vaccine and CPV2 Canigen C3 Vaccine viruses^20^. Sequences were aligned using Clustal-W^44^ or in some cases for codon alignments using Muscle^45^. The Maximum likelihood (ML) method with the best fitting model, as determined by MEGA, was selected for generating phylogenetic trees and the robustness of different nodes assessed by bootstrap analysis using 1000 replicates for nucleotide alignments and 500 replicates for amino acid alignments.

### SimPlot analysis

Similarity plot (SimPlot) was used to observe possible recombination between our sequence and related sequences. A 200 nucleotides sliding window at 20 nucleotides intervals and the F84 distance model with maximum likelihood method was used. Percentage identities at each analysis point were plotted on a line chart^46^.

### Canine astrovirus partial open reading frame 1b (ORF1b) PCR and Sanger Sequencing

Real time PCR targeting the ORF1b region of canine astrovirus was conducted in order to see if the individual faecal samples were positive or negative for canine astrovirus. Primers (Micromon - Monash University, Clayton, Victoria, Australia) were designed to amplify RNA dependent RNA Polymerase gene (RdRP) encoded by ORF1b using Primer Blast and OligoCalc software^47,48^. TRB-CAstV ORF1b-F3 (5′-TCCATGGGCCTAACAAGCAG-3′) and TRB-CAstV ORF1b-R3 (5′-TGGTTTGAGAAGTGAGGCCA-3′) were used as forward and reverse primer respectively. cDNA was synthesized as described above. The PCR master mix was made with PowerUp SYBR Green Master Mix (Thermofisher Scientific) with 1 mM of each primer and 2 ul of cDNA in a total reaction volume of 10 ul. A QuantStudio™ Flex 6 real-time thermal cycler (Applied Biosystems) was used for amplification at 95 °C for 10 min, 40 cycles of 95 °C for 30 sec, 54 °C annealing temperature for 30 sec, 72 °C for 30 sec and a final 72 °C step for 3 min. A melt curve analysis was performed immediately post PCR with reactions subjected to 95 °C for 15 sec, then 60 °C for 1 min followed by a continuous temperature ramp between 60 °C and 95 °C increasing at 0.05 °C/sec. PCR products of ~269 bp size were purified using the 2% Size Select E-Gel System (Thermo Fisher, Waltham, MA, USA). These products were re-amplified by AmpliTaq Gold™ 360 Master Mix (Applied Bio systems) using the same primers and PCR program mentioned above. PCR products were sequenced using the Big Dye Terminator Cycle v3.1 on a Hitachi 3500xl genetic analyser (Applied Biosystems, Foster City, California, USA)^49^.

## Supplementary information

Supplementary Information

## Data Availability

All the sequences generated have been deposited in NCBI Genbank with accession numbers MK026166 and MK205369 - MK205391. Additional datasets analysed in the paper can be made available from the authors upon reasonable request.

## References

[1] Steiner, J. M. In *BSAVA manual of canine and feline gastroenterology* 13–21 (BSAVA Library, 2005).

[2] da Rocha Gizzi AB (2014). Presence of infectious agents and co-infections in diarrheic dogs determined with a real-time polymerase chain reaction-based panel. BMC veterinary research.

[3] Alves, C. D. *et al*. Identification of enteric viruses circulating in a dog population with low vaccine coverage. *Brazilian Journal of Microbiology* (2018). 10.1016/j.bjm.2018.02.006 PMC6175709 29588198

[4] Decaro N (2011). Western European epidemiological survey for parvovirus and coronavirus infections in dogs. The Veterinary Journal.

[5] Martella V (2011). Detection and characterization of canine astroviruses. Journal of General Virology.

[6] Toffan A (2009). Genetic characterization of a new astrovirus detected in dogs suffering from diarrhoea. Veterinary microbiology.

[7] Zhu A (2011). Isolation and characterization of canine astrovirus in China. Archives of virology.

[8] Cavalli A (2014). Detection and genetic characterization of Canine parvovirus and Canine coronavirus strains circulating in district of Tirana in Albania. Journal of Veterinary Diagnostic Investigation.

[9] Wilkes RP (2014). Rapid and sensitive detection of canine distemper virus by one-tube reverse transcription-insulated isothermal polymerase chain reaction. BMC veterinary research.

[10] Ortega AF, Martínez-Castañeda JS, Bautista-Gómez LG, Muñoz RF, Hernández IQ (2017). Identification of co-infection by rotavirus and parvovirus in dogs with gastroenteritis in Mexico. brazilian journal of microbiology.

[11] Blomström A-L (2011). Viral metagenomics as an emerging and powerful tool in veterinary medicine. Veterinary Quarterly.

[12] Somerville LK, Ratnamohan VM, Dwyer DE, Kok J (2015). Molecular diagnosis of respiratory viruses. Pathology.

[13] Li L (2011). Viruses in diarrhoeic dogs include novel kobuviruses and sapoviruses. Journal of General Virology.

[14] Moreno PS (2017). Characterisation of the canine faecal virome in healthy dogs and dogs with acute diarrhoea using shotgun metagenomics. PloS one.

[15] Caddy SL, Goodfellow I (2015). Complete genome sequence of canine astrovirus with molecular and epidemiological characterisation of UK strains. Veterinary microbiology.

[16] Alves CDBT (2018). Mamastrovirus 5 detected in a crab-eating fox (Cerdocyon thous): Expanding wildlife host range of astroviruses. Comparative Immunology, Microbiology and Infectious Diseases.

[17] Mihalov-Kovács E (2017). Genome analysis of canine astroviruses reveals genetic heterogeneity and suggests possible inter-species transmission. Virus research.

[18] Alves, C. D. *et al*. Detection and genetic characterization of Mamastrovirus 5 from Brazilian dogs. *Brazilian Journal of Microbiology* (2018). 10.1016/j.bjm.2017.09.008 PMC6066731 29456114

[19] Parrish CR (1991). Mapping specific functions in the capsid structure of canine parvovirus and feline panleukopenia virus using infectious plasmid clones. Virology.

[20] Meggiolaro MN (2017). MT-PCR panel detection of canine parvovirus (CPV-2): Vaccine and wild-type CPV-2 can be difficult to differentiate in canine diagnostic fecal samples. Molecular and cellular probes.

[21] Munday JS, Dunowska M, Laurie RE, Hills S (2016). Genomic characterisation of canine papillomavirus type 17, a possible rare cause of canine oral squamous cell carcinoma. Veterinary microbiology.

[22] Lange CE, Tobler K, Lehner A, Vetsch E, Favrot C (2012). A case of a canine pigmented plaque associated with the presence of a Chi‐papillomavirus. Veterinary dermatology.

[23] Tobler, K., Lange, C., Ackermann, M. & Favrot, C. Canine papillomavirus - 4 isolate pug2006, complete genome, https://www.ncbi.nlm.nih.gov/nuccore/EF584537 (2007).

[24] Bourke B, Chan VL, Sherman P (1998). Campylobacter upsaliensis: waiting in the wings. Clinical microbiology reviews.

[25] Lew-Tabor. Overview of Enteric Campylobacteriosis, https://www.merckvetmanual.com/digestive-system/enteric%20campylobacteriosis/overview-of-enteric-campylobacteriosis (2018).

[26] Preston M (1990). *In vitro* susceptibility of “Campylobacter upsaliensis” to twenty-four antimicrobial agents. European Journal of Clinical Microbiology and Infectious Diseases.

[27] Choi S (2014). Phylogenetic analysis of astrovirus and kobuvirus in Korean dogs. Journal of Veterinary Medical Science.

[28] Castro, T. *et al*. Molecular characterisation of calicivirus and astrovirus in puppies with enteritis (2013). 10.1136/vr.101566 23605075

[29] Takano T, Takashina M, Doki T, Hohdatsu T (2015). Detection of canine astrovirus in dogs with diarrhea in Japan. Archives of virology.

[30] Grellet A (2012). Prevalence and risk factors of astrovirus infection in puppies from French breeding kennels. Veterinary microbiology.

[31] Jeong HS, Jeong A, Cheon D-S (2012). Epidemiology of astrovirus infection in children. Korean journal of pediatrics.

[32] Méndez-Toss M (2004). Prevalence and genetic diversity of human astroviruses in Mexican children with symptomatic and asymptomatic infections. Journal of clinical microbiology.

[33] Maldonado Y (1998). Population-based prevalence of symptomatic and asymptomatic astrovirus infection in rural Mayan infants. Journal of infectious diseases.

[34] Australian Government, D. O. A. A. W. R. Importing frozen canine semen from approved countries, http://www.agriculture.gov.au/cats-dogs/step-by-step-guides/canine-semen-approved-countries#step-2-confirm-general-eligibilitytimeframe-before–starting-the-export-process (2018).

[35] Thomassen R, Farstad W (2009). Artificial insemination in canids: a useful tool in breeding and conservation. Theriogenology.

[36] Arias, E. M. A. C. F. In Fields Virology Vol. I (eds David, M. Knipe and Peter, M. Howley) 982–1000 (Philadelphia: Lippincott Williams & Wilkins, 2007).

[37] Johnson C, Hargest V, Cortez V, Meliopoulos V, Schultz-Cherry S (2017). Astrovirus pathogenesis. Viruses.

[38] Vibin, J. *et al*. Metagenomics detection and characterisation of viruses in faecal samples from Australian wild birds. *Scientific reports* **8** (2018). 10.1038/s41598-018-26851-1 PMC5989203 29875375

[39] Altschul SF, Gish W, Miller W, Myers EW, Lipman DJ (1990). Basic local alignment search tool. Journal of molecular biology.

[40] Mount DW (2007). Using the basic local alignment search tool (BLAST). Cold Spring Harbor Protocols.

[41] Caboche S, Audebert C, Lemoine Y, Hot D (2014). Comparison of mapping algorithms used in high-throughput sequencing: application to Ion Torrent data. BMC genomics.

[42] Thorvaldsdóttir H, Robinson JT, Mesirov JP (2013). Integrative Genomics Viewer (IGV): high-performance genomics data visualization and exploration. Briefings in bioinformatics.

[43] Kumar S, Stecher G, Tamura K (2016). MEGA7: molecular evolutionary genetics analysis version 7.0 for bigger datasets. Molecular biology and evolution.

[44] Larkin MA (2007). Clustal W and Clustal X version 2.0. bioinformatics.

[45] Edgar RC (2004). MUSCLE: multiple sequence alignment with high accuracy and high throughput. Nucleic acids research.

[46] Martin D, Posada D, Crandall K, Williamson C (2005). A modified bootscan algorithm for automated identification of recombinant sequences and recombination breakpoints. AIDS Research & Human Retroviruses.

[47] Ye J (2012). Primer-BLAST: a tool to design target-specific primers for polymerase chain reaction. BMC bioinformatics.

[48] Kibbe WA (2007). OligoCalc: an online oligonucleotide properties calculator. Nucleic acids research.

[49] Chamings A (2018). Detection and characterisation of coronaviruses in migratory and non-migratory Australian wild birds. Scientific reports.

[50] Nei, M. & Kumar, S. *Molecular evolution and phylogenetics*. (Oxford university press, 2000).

[51] Jones DT, Taylor WR, Thornton JM (1992). The rapid generation of mutation data matrices from protein sequences. Bioinformatics.

[52] Tamura K, Nei M (1993). Estimation of the number of nucleotide substitutions in the control region of mitochondrial DNA in humans and chimpanzees. Molecular biology and evolution.

