# Supplementary material for: Detection and characterisation of canine astrovirus, canine parvovirus and canine papillomavirus in puppies using next generation sequencing
Source: Sci Rep. 2019 Mar 14;9:4602. doi: 10.1038/s41598-019-41045-z (PMC6418273; doi:10.1038/s41598-019-41045-z)
Supplement: Supplementary file 1 — Supplementary Information [file 41598_2019_41045_MOESM1_ESM.pdf]

Detection and characterisation of canine astrovirus, canine parvovirus and canine papillomavirus in puppies using next generation sequencing

Tarka Raj Bhatta[1,2], Anthony Chamings [1,2], Jessy Vibin [1,2] & Soren Alexandersen*[1,2,3]

[1]Geelong Centre for Emerging Infectious Diseases, Geelong, VIC 3220, Australia; [2]Deakin University, School of Medicine, Geelong, VIC 3220, Australia; [3]Barwon Health, University Hospital Geelong, Geelong, VIC 3220 Australia

*Corresponding Author: soren.alexandersen@deakin.edu.au

Supplementary Table S1. Comparison of the canine astrovirus near complete sequence of DF-BC15- CAV-AUS-2017 with selected reference sequences from Genbank.

Virus Sequence from dog samples	NCBI reference sequence	Nucleotide match	Identity %
DF-BC15-CAV-AUS-2017	KP404149-CAV-Gillingham-UK-2012	6260/6611	94.69
DF-BC15-CAV-AUS-2017	KX599350-CAV-6-HUN-2012	6169/6576	93.81
DF-BC15-CAV-AUS-2017	KX599351-CAV-115-HUN-2012	5848/6563	89.11
DF-BC15-CAV-AUS-2017	KX599353-CAV-135-HUN-2012	5811/6565	88.51
DF-BC15-CAV-AUS-2017	KX599349-CAV-2-HUN-2012	5800/6566	88.33
DF-BC15-CAV-AUS-2017	KY765684-CTAV-BRA-2015	5762/6559	87.85
DF-BC15-CAV-AUS-2017	KX756441-CAV-DD1-AUS-2012	5702/6507	87.62
DF-BC15-CAV-AUS-2017	KP404150-CAV-Lincoln-UK-2012	5762/6583	87.53

Supplementary Table S2. Comparison of the ORF1a sequence of DF-BC15-CAV-AUS-2017 with selected reference sequences from Genbank

Canine astrovirus ORF1a sequence from dog samples	NCBI reference sequence	Nucleotide match	Identity %	Amino acid match %
DF-BC15-CAV-ORF1a-AUS-2017	KX599350-CAV-6-HUN-2012	2572/2670	96.32	879/889 (98.87%)
DF-BC15-CAV-ORF1a-AUS-2017	KX599351-CAV-115-HUN-2012	2559/2670	95.84	876/889 (98.54%)
DF-BC15-CAV-ORF1a-AUS-2017	KP404149-CAV-Gillingham-UK-2012	2549/2670	95.46	882/889 (99.21%)
DF-BC15-CAV-ORF1a-AUS-2017	KX756441-CAV-DD1-AUS-2012	2501/2670	93.67	876/889 (98.54%)
DF-BC15-CAV-ORF1a-AUS-2017	KP404150-CAV-Lincoln-UK-2012	2478/2670	92.81	872/889 (98.08%)

Supplementary Table S3. Comparison of the seven different regions of the canine parvovirus sequence generated by NGS with the reference sequence (M38245) from Genbank.

**Regions of DF-BC16-CPV-AUS-2017**	**Reference sequence**	**Query Cover %**	**Nucleotide match**	**Identity %**	**Nucleotide differences**	**Amino acid match %**
Non-coding-NS1 (163-486)	M38245-CPV-b-USA-1990	100	324/324	100	-	71 /71 (100%)
NS1 (631-1083)	M38245-CPV-b-USA-1990	100	452/453	99.8	A-G at 941	150/150 (100%)
NS1 (1844-2162)	M38245-CPV-b-USA-1990	100	318/319	99.7	A-G at 2024	106/106 (100%)
VP1, splice (2273-2730)	M38245-CPV-b-USA-1990	100	457/458	99.8	G-A at 2341 in intron	124/124 (100%)
VP2 (2886-3450)	M38245-CPV-b-USA-1990	100	563/565	99.6	A-T at 3045<br>T-C at 3088	186/188 (99%)
VP2 (3593-4213)	M38245-CPV-b-USA-1990	100	615/621	99.0	T-G at 3675<br>C-G at 3685<br>G-T at 3699<br>T-A at 3749<br>A-G at 3909<br>A-G at 4062	200/206 (97%)
Non-coding (4711-4850)	M38245-CPV-b-USA-1990	100	139/140	99.3	T-C at 4789	Non-coding

Supplementary Table S4. Comparison of the eight different sequences obtained from four regions of canine papillomavirus 17 sequence generated by NGS with the reference sequence (KT272399) from Genbank.

Region of DS-CPapV-AUS-2017	Reference sequence	Nucleotide match	Identity %	Nucleotide differences	Amino acid match %
E1 (750-957)	KT272399-CpapV-17-NZ-2014	208/208	100	-	69/69 (100%)
E2 (2963-3161)	KT272399-CpapV-17-NZ-2014	199/199	100	-	66/66 (100%)
Bet. E2 & L2 (4070-4360)	KT272399-CpapV-17-NZ-2014	291/291	100	-	Non-Coding
L2 (4572-4784)	KT272399-CpapV-17-NZ-2014	213/213	100	-	70/70 (100%)
L2 (4822-5022)	KT272399-CpapV-17-NZ-2014	200/201	99.5	G-A at 4909	66/66 (100%)
L2 (5616-5826)	KT272399-CpapV-17-NZ-2014	210-211/211	99.5-100	C/T at 5695	69/69 (100%)
L2, NCR, L1 (5872-6198)	KT272399-CpapV-17-NZ-2014	326/327	99.6	G-A at 6173	L2: 37/37 (100%) L1:65/65 (100%)
L1 (6625-7032)	KT272399-CPapV-17-NZ-2014	406/408	99.5	A-G at 6698 T-C at 6968	135/135 (100%)

Supplementary Table S5. Comparison of the different regions of canine papillomavirus 4 and 8 sequences generated by NGS with reference sequences (EF584537) and (HQ262536) respectively from Genbank.

Sequence name	Region	Reference sequence	Nucleotide match	Identity %	Amino acid match %
DS-BC18-CPapV-4-AUS-2017	E7 (634-773)	EF584537-CPapV-4-Swiss-2007	140/140	100	46/46 (100%)
DS-BC16-CPapV-4-AUS-2017	E1 (1678-1844)	EF584537-CPapV-4-Swiss-2007	167/167	100	55/55 (100%)
DS-BC16-CPapV-4-AUS-2017	E2 (3993-4106)	EF584537-CPapV-4-Swiss-2007	114/114	100	37/37 (100%)
DS-BC16-CPapV-4-AUS-2017	L2 (4682-4943)	EF584537-CPapV-4-Swiss-2007	261/262	99.6 A --- T at 4749	86/86 (100%)
DS-BC18-CPapV-8-AUS-2017	E1 (758-981)	HQ262536-CPapV-8-Swiss-2010	224/224	100	74/74 (100%)
DS-BC18-CPapV-8-AUS-2017	E2 (2898-3292)	HQ262536-CPapV-8-Swiss-2010	395/395	100	131/131 (100%)
DS-BC18-CPapV-8-AUS-2017	L1 (6109-6392)	HQ262536-CPapV-8-Swiss-2010	284/284	100	94/94 (100%)

Supplementary Table S6. **The virus sequences from reference Genbank used for initial mapping of NGS reads.**

Sample	Virus	Genbank reference sequence used for TMAP
DF-1-2-AUS-2017	Canine astrovirus	KP404149-CAV-Gillingham-UK-2012
DF-1-2-AUS-2017	Canine parvovirus	M38245-CPV-b-USA-1990
DS-3-4-5-6-AUS-2017	Canine papillomavirus 17	KT272399-CPapV-17-NZ-2014
DS-4-AUS-2017	Canine papillomavirus 4	EF584537-CPapV-4-Swiss-2007
DS-6-AUS-2017	Canine papillomavirus 8	HQ262536-CPapV-8-Swiss-2010
